# Comprehensive early intervention for patients with first-episode psychosis in Japan (J-CAP): study protocol for a randomised controlled trial

**DOI:** 10.1186/1745-6215-12-156

**Published:** 2011-06-20

**Authors:** Shinsuke Koike, Atsushi Nishida, Syudo Yamasaki, Kayo Ichihashi, Sanae Maegawa, Tatsunobu Natsubori, Hirohiko Harima, Kiyoto Kasai, Izumi Fujita, Masanori Harada, Yuji Okazaki

**Affiliations:** 1 Department of Neuropsychiatry, Graduate School of Medicine, The University of Tokyo, Bunkyo-ku, Tokyo, 113-8655, Japan; 2 Department of Psychiatry & Behavioral Science, Tokyo Metropolitan Institute of Medical Science, Setagaya-ku, Tokyo, 156-8506, Japan; 3 Tokyo Metropolitan Matsuzawa Hospital, Setagaya-ku, Tokyo, 156-0057, Japan; 4 Department of Rehabilitation, The University of Tokyo Hospital, Bunkyo-ku, Tokyo, 113-8655, Japan; 5 Hinaga General Center for Mental Care and Sasagawa Clinic, Yokkaichi-shi, Mie, 510-8575, Japan; 6 Mie Psychiatric Mental Care Center, Tsu-shi, Mie, 514-0818, Japan

## Abstract

**Introduction:**

Comprehensive approaches for patients with psychotic symptoms play essential roles in the symptomatic and functional outcomes of patients, especially during disease onset. In Japan, the shortage of mental health services, particularly for outpatients, and community-based supports has been a major problem. The purpose of this trial is to investigate the effectiveness and affordability of 18-month comprehensive early intervention services for patients with first-episode psychosis compared with typical treatment.

**Methods:**

This interventional, parallel, single-blinded (open but blinded raters trial) was effectively designed. The participants are patients with a diagnosis of F2 or F3 (International Classification of Disease, 10 th revision), with psychotic symptoms. The inclusion criteria were an age of 15-35 years, onset of psychotic symptoms within 5 years, first-episode psychosis, and residence in the catchment area of each site. Allocation will be conducted equally between case management and standard care groups. After enrollment, standard care will be provided for both groups, and community-based care to promote recovery for 18 months will be provided for the comprehensive approach group. The primary outcome will be the function domain of the global assessment of functioning scores at 18 months after enrollment. Data assessment will be performed at enrollment and 18, 36, and 60 months after enrollment. The target sample size will be 150, and registration will occur from March 1, 2011, to September 30, 2012.

**Discussion:**

This trial will provide promising results about the effectiveness and cost-effectiveness of early intervention services in Japan to improve the quality and quantity of community mental health services.

**Trial registration:**

This trial was registered in The University Hospital Medical Information Network Clinical Trials Registry (No. UMIN000005092).

## Introduction

Comprehensive approaches for patients with schizophrenia play essential roles in their symptomatic and functional outcomes[1-4]. Particularly after the onset of psychosis, most patients and their families are extremely confused and distressed because of the conditions of patients and the lack of knowledge about their illnesses. Several randomized controlled trials (RCTs) have suggested that intensive community-based care for patients with first-episode psychosis (FEP) improve patient symptoms[5,6] and functional outcomes such as relapse, readmission, dropout from services, and social and vocational functioning[5-8]. Furthermore, additional analyses of cost-effectiveness revealed that patients with FEP who received intensive care had better outcomes without increase costs[9]. On the basis of these results, the use of early intervention services (EIS) has increased, especially in England[1].

Japan has among the best medical services in the world (e.g., the longest average life expectancy and smallest perinatal mortality rate). However, the shortage of mental health services for outpatients has been a major problem. In Japan, there are approximately 350,000 psychiatric beds, and approximately 210,000 patients majorly diagnosed with schizophrenia remain in psychiatric hospitals for more than 1 year (Reported from the Minster of Health, Labour, and Welfare). One issue preventing the discharge of patients with severe mental illnesses from hospitals is the shortage of community mental health services with psychosocial approaches, including family support despite the vast medical resources of Japan[10]. To change this situation and broaden EIS based on community settings, an RCT is needed to confirm the clinical effectiveness and affordability of EIS compared with existing hospital-based mental health service systems in Japan. Additionally, these trials must examine whether these specialized community mental health services will sustain the functional recovery of patients even after they finish 18-month support.

## Methods

### Trial design

**Figure 1:**
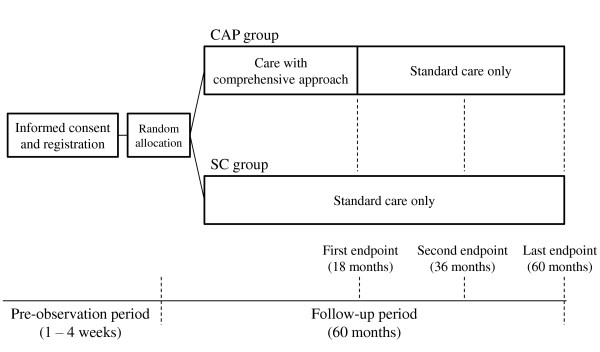
**Entire design of this trial**.

This trial is designed with effectiveness, interventional, parallel, single-blinded (open but blinded raters) trial. The allocation of participants will be equal (1:1) between the comprehensive approach (CAP) and standard care (SC) groups. The entire trial design is illustrated in Figure 1. After enrollment, all participants will be provided standard care, and in the CAP group, comprehensive community-based care by an early intervention team will be provided for 18 months. Eighteen months after enrollment, all participants will be provided standard care only. The assessment of clinical data will be conducted at enrollment and 18 (first end point), 36 (second end point), and 60 months after enrollment (last end point). The target sample size will be 150, and registration will occur from March 1, 2011, to September 30, 2012. The last follow-up date will be September 30, 2017. This trial was registered in the University Hospital Medical Information Network Clinical Trials Registry (UMIN-CTR) accepted from the International Committee of Medical Journal Editors (ICMJE) (No. UMIN000005092).

### Participants

The participants are patients who received a diagnosis of F2 or F3 (International Classification of Disease, 10 th revision)[11], with psychotic symptoms, at 4 sites: the University of Tokyo Hospital, Tokyo Metropolitan Matsuzawa Hospital, Mie Prefectural Mental Medical Center, and Hinaga General Center for Mental Care and Sasagawa Clinic. The relevant details of the sites are summarized in Table 1.

**Table 1:** Characteristics of facilities, number of patients, and number of staff members on early intervention teams at each site

		Univ of Tokyo	Matsuzawa	Mie	Hinaga
Beds in closed wards		29	713	200	255

Beds in open wards		31	149	200	300

Annual	Total	1107	2150	896	706
	
hospital	15-35 years old	381	445	165	184
	
admissions	F2	129	212	55	94

Average number of daily patients the in outpatient unit		170	329.4	214.2	309.8

Average number of daily participants in the outpatient rehabilitation center		33.1	57.6	49.9	109.6

Annual new patients	Total	1102	5656	1405	1150
	
	15-35 years old	474	NA	476	450
	
	F2	124	NA	43	58

Catchment area		Bunkyo, Arakawa, Taito, and Chiyoda	Setagaya, Suginami, Ota, Meguro, Shibuya, Komae, Chofu, and Mitaka	Tsu, Matsuzaka, Suzuka, Iga, Nabari, and Ise	Yokkaichi, Suzuka, Kameyama, and Mie-gun

Population (× 1000)		800	3020	870	620

	Psychiatrists	6	5	1	3
	
	Nurses	0	4	5	6
	
	Psychologists	3	0	1	2
	
Members of the EIS team	Psychiatric social workers	1	2	3	2
	
	Vocational workers	0	0	1	1
	
	Pharmacist	0	0	1	0

Abbreviations: Univ of Tokyo, the University of Tokyo Hospital; Matsuzawa, Tokyo Metropolitan Matsuzawa Hospital; Mie, Mie Prefectural Mental Medical Center; Hinaga, Hinaga General Center for Mental Care and Sasagawa Clinic; EIS, early intervention service.

Eligibility criteria are summarized in Table 2. The inclusion criteria are an age of 15-35 years old, onset of psychotic symptoms within 5 years, first-episode psychosis, and residence in the catchment area of each site (Table 1). Psychotic symptoms are defined by the first clear evidence of a positive psychotic symptom (i.e., delusion, hallucination, or thought disorder) that was scored 4 or higher on the positive and negative symptom scales (PANSS) [12] regardless of its duration.

**Table 2:** Summary of eligibility criteria.

Inclusion criteria:

1. Residence in the catchment area of each site (also see Table 1)

2. Age between 15 and 35 years old

3. Within 5 years of the onset of psychotic symptoms

4. FEP



Exclusion criteria:

1. Premorbid IQ below 80

2. Unable to sufficiently communicate in Japanese

3. Require care for any organic mental disorder

4. Require inpatient care for any physical condition

5. A history of dependency on alcohol and/or other substance of abuse

6. Under physical restraint and/or seclusion

7. Received electroconvulsive therapy and/or transcranial magnetic stimulation therapy within the past month

8. Present involuntary hospitalization

9. Not having received an explanation of his or her condition or diagnosis by a psychiatrist

10. Regarded as inappropriate by medical doctors in charge for any other reason

Target patients: Patients with a diagnosis of F2 and F3 (International Classification of Disease, 10 th revision), with psychotic symptoms.

Exclusion criteria are premorbid IQ [13,14] of less than 80, inability to sufficiently communicate in Japanese, requirement of care for any organic mental disorder or inpatient care for any physical condition, a history of dependency on alcohol and/or other substance of abuse, under physical restraint and/or seclusion, received electroconvulsive therapy and/or transcranial magnetic stimulation therapy within the past month, present involuntary hospitalization, not having been given the explanation of his or her condition or diagnosis from a psychiatrist, and being regarded as inappropriate by their doctors for any other reason. Although the use of illegal substances such as cannabis among young people has been a major problem, a very small number of young people have used these drugs in Japan. Therefore, we adopted a history of continuous substance abuse as one of the exclusion criteria in this trial.

All of the eligibility criteria will be assessed by psychiatrists at each site; to complement these criteria, the laboratory data of all patients will be assessed within 30 days of enrollment, and a brain CT or MRI will be performed within 12 months.

### Ethical consideration

All participants will be presented with written informed consent to the ethical committee of each site (Univesity of Tokyo, No. 3307; Matsuzawa, No. 22-23; Mie, H23.2.21; Hinaga, H22.12.22) according to the Declaration of Helsinki after receiving a complete explanation of this trial.

### Intervention

For the CAP group, we will provide specialized early intervention teams by well-trained case managers and psychiatrists about their symptoms and daily activities for 18 months after enrollment. Case managers will promote participant recovery and social participation in cooperation with the early intervention team by using combination of cognitive behavioral therapeutic (CBT) approaches, psychoeducational approaches, family interventions, discharge support, and pharmacological therapy in accordance with the guidelines for FEP. The details of comprehensive community-based care are described later in the text. After 18 months, the participants will receive only standard care at each site. For the SC group, we will provide only standard care at each site throughout the entire study.

#### CBT approach

Several researches have suggested that CBT adapted to patients with early psychosis has effectiveness regarding their persistent positive symptoms, anxiety, and/or depression[15]. In this trial, we will not adopt structured CBT but needs-based CBT approaches in comprehensive community-based care. Case managers will receive basic CBT training and use the skills and techniques of CBT in comprehensive community-based care. The range and intensity of CBT will vary according to participant need as assessed by the early intervention team. Through CBT sessions, case managers will provide monitoring and coping skills for patients regarding their symptoms as well as functional skills for their daily lives.

#### Psychoeducational approach

Young people and their families rarely learn about symptoms, outcomes, and methods of coping with psychosis until they have mental illnesses; in other cases, they received incorrect information in addition to being subjected to discrimination and stigma. Therefore, they have significant stress and confusion because of the lack of information about psychosis. Several studies have suggested that psychoeducation is effective for relapse prevention, reducing hospital admission, and adherence of medication[16-18]. In this trial, the psychoeducational program for people with FEP will consist of sessions that provide not only information about etiology, symptoms, treatments, and outcome of psychosis, but also positive and helpful information toward functional recovery. In addition, we will provide psychoeducational programs for their families as described in the following section.

#### Family intervention

Family interventions will consist of 2 main domains of psychoeducation and family support for the family members of patients with mental illnesses. As described above, family members usually have little correct knowledge but a plethora of incorrect information and a great deal of stigma. Therefore, family members are also confused about methods of assisting patients who have recently exhibited psychotic symptoms. Several studies have suggested that family intervention for early psychosis is effective for relapse prevention, reducing hospital admission, and adherence of medication[19-21]. Family interventions for early psychosis will consist of psychoeducational programs and psychological therapy or counseling for families to relieve their burdens regarding the care of people with psychosis. Psychoeducational programs for families will consist of lectures about psychosis and peer group sessions. Psychological therapy and counseling for families will be provided through individual sessions.

#### Discharge support

More than 210,000 patients with schizophrenia stay in hospital for more than 1 year in Japan, mainly because of a lack of community mental health services[10]. This is particularly true for patients with psychosis for whom their family members are unable to obtain information and support from psychiatric community services. In this trial, case managers will encourage patients to live in their communities by introducing additional psychiatric community services, if needed.

#### Pharmacological therapy in accordance with the guidelines for FEP

Several pharmacological guidelines for schizophrenia and mood disorder are available worldwide. However, some psychiatrists in Japan have developed experimence-oriented strategies that result in polypharmacy for patients with schizophrenia[22]. Polypharmacy can exacerbate the negative symptoms and cognitive impairments of patients in addition to promoting dropped-out from treatment[22]. One reason why psychiatrists tend to prescribe more tablets may be that patients' treatment strategy and prescription are mainly decided by their psychiatrists who know little information except from the consultation with patients and their family members. In this trial, we will conduct regular meetings with early intervention teams and propose treatment and prescription strategies to psychiatrists according to the guidelines to eliminate unnecessary prescriptions.

#### Strategy for replacement from EIS

Recent studies about EIS for patients with FEP had negative results regarding the effectiveness of EIS discontinued in a longer term[23,24]. One reason may be that it is difficult to prepare patients to finish EIS and switch to normal services. On the basis of this result, we will emphasize "graduating from the EIS" and provide the policy of EI services to switch over to usual community services in order to easily maintain the effectives of EIS. Thus, we will also investigate whether this strategy for switch over will prolong the effectiveness of EIS.

#### Supervision for case management

To standardize the intensity and quality of EIS, we will conduct training courses at least twice a year with required participation by case managers at each site, at which they will have to discuss their practices and cases. Supervision in meetings will be provided, and professionals and supervisors of other services will discuss and provide advice about their practices and cases.

### Outcomes and measurement items

We will adopt the function domain of the global assessment of functioning (GAF-F) [25] scores at the first end point as the primary outcome measure. Secondary outcomes will be GAF-F at the second and last end points, symptom domain of global assessment of functioning (GAF-S), PANSS,[12] the World Health Organization quality of life 26-item version (WHO-QOL26),[26,27] brief evaluation of medication influences and beliefs (BEMIB),[28] care satisfaction of participants and their families, educational and vocational recovery rates, remission rate, re-admission rate, lost to follow-up rate, self-harm and suicide attempt rate, suicide rate, engagement behavior, and direct and indirect costs at each end point. All measurement items will also be assessed at baseline. We will record the presence of comorbid mental and physical disorders and relevant sociodemographic and clinical information about the participants and their families at baseline and at each end point. The details of the assessments are discussed in the following sections.

#### GAF, GAF-F, and GAF-S

GAF records the current objective symptomatic and functional conditions of participants on one analogous scale ranging from 0 (poor) to 100 (good)[25]. GAF-F rates the social and occupational functions of patients, and GAF-S rates their symptoms. GAF is adopted as the worse score of GAF-F and GAF-S. We will also use the modified GAF scale [29] to measure GAF, GAF-F, and GAF-S.

#### PANSS

PANSS records the current objective symptoms of patients on 30 items[12]. PANSS consists of 3 domains: positive symptoms, negative symptoms, and general psychopathology. Each item is rated from 1 (absent) to 7 (extreme), and the total score ranges from 30 to 210.

#### WHO-QOL26

WHO-QOL26 [26,27] measures the current subjective satisfaction of participants regarding their quality of life on 26 items. WHO-QOL26 consists of 4 domains: physical health, psychological health, social relationships, and environment. Each item is rated from 1 (poor) to 5 (good) and presented as an average score.

#### BEMIB

BEMIB measures the current drug adherence of participants to their medications on 8 items[28]. Each item is rated from 1 (completely disagree) to 5 (completely agree), and the total score ranges from 8 to 40.

#### Care satisfaction

Care satisfaction will be measured by one simple item rated from 1 (very satisfied) to 4 (very dissatisfied). Participants and their families will provide subjective care satisfaction ratings after enrollment.

#### Recovery rate

Recovery will be measured by using the definition sheet adopted for EIS in England[30]. The item in the sheet termed "TRAINING AND OCCUPATION," which is rated from 0 (employment) to 4 (Not in Education, Employment or Training; NEET), will be assessed. For the initial assessment, this rating will be taken from the best occupational status achieved within the last 6 months for each end point taken from the point of last assessment.

#### Remission rate

We defined remission using a proposal from the Remission in Schizophrenia Working Group [31] that defined symptomatic remission of illness using 8 corresponding PANSS subscores (P1, P2, P3, N1, N4, N6, G5, and G9) of mild or less simultaneously on all items and for which remission regarding these scores was maintained for at least 6 months.

#### Re-admission rate

We will record all voluntary and involuntary admissions of participants at registration and during the follow-up period.

#### Lost to follow-up rate

Lost to follow-up will be defined at each end point as the refusal of further treatment despite the need and several attempts of reengagement (phone calls to patients and families in both groups and home visits to participants in the CAP group)[32]. We will consider the last successful contact the date of lost to follow-up.

#### Self-harm, suicide attempt, and suicide rate

Self-harm and suicide attempt will be measured using the items in the definition sheet "SELF-HARM and SUICIDE" on a scale of 0 (None) to 4 (Severe problems)[30]. We will assess the inquiry sheets for all of the suicide and severe self-harm actions at all sites.

#### Engagement behavior

Engagement, family engagement, and social relationships will be measured using the items in the definition sheet "ENGAGEMENT, FAMILY/CARER ENGAGEMENT, and RELATIONSHIPS" on a scale of 0 (Severe problems) to 4 (Very good) [30].

#### Service costs and cost-effectiveness

Service costs will be calculated using the Client Service Receipt Inventory (CSRI),[9,33], which can estimate service costs and cost-effectiveness, particularly in psychiatric contexts. On the basis of the CSRI, we will conduct interviews of daily living (e.g., service use, school, employment, and family conditions) at registration, at each end point, and if possible, at intermediate points between end points. Service costs will be calculated by the service costs and appropriate local unit costs. Cost-effectiveness will be calculated by the combination of the outcomes related to the service costs and participant subjective quality of life assessments using WHO-QOL26. Unavailable cost data will be estimated using other resources and models.

### Data reliability

Some methodological problems regarding interrater reliability will exist. To control the quality of data assessment, we will conduct lectures for PANSS and GAF, including GAF-F and GAF-S, more than once a year using video movies to ensure accurate scoring. We will also calculate interrater reliability for PANSS and GAF scores provided by the raters in the lecture, and we will provide feedback for these results to maintain a high quality of assessment.

### Sample size

The planned number of participants is 150, as determined by previous RCT results, the clustering effects of each site, and the possibility of exhaustion of our resources. An RCT of EIS for patients with FEP in Holland indicated that EIS improved participant GAF-F scores in 24 months[5]. Another RCT of CBT in England revealed that CBT improved patient readmission rates and GAF scores in 18 months[6,7]. The effect sizes calculated from their results were 0.26, 0.46, and 0.58, respectively. The estimated sample sizes using G*Power 3[34] (alpha error = 0.05, beta error = 0.2) were 370, 150, and 96, respectively.

We will adopt 1 interim analysis and consider stopping the trial if the participants in the CAP group have unexpected effective outcomes compared with those in the SC group. We will conduct the interim analysis when half of the participants finish the trials until the first end point. Stopping rules will be planned on the basis of the O'Brien-Fleming method [35] for the GAF-F score, re-admission rate, lost to follow-up rate, self-harm and suicide attempt rate, and suicide rate at the first end point. We will consider stopping the trial on the basis of the stopping rule, baseline data, missing data, and site. Because of ethical issues, we will provide EIS to all participants until 18 months after allocation if the trial is halted.

### Randomization

Enrollment and random allocation will be performed by central registration at the University Hospital Clinical Trial Alliance Clinical Research Supporting System (UHCT ACReSS) at the University of Tokyo. The managers of each site will enroll participants after examining their eligibility and informed consent. The type of allocation will be stratified and block randomization. We will adopt stratification as sites and inpatients/outpatients at enrollment. Owing to allocation concealment, the block size will be provided by UHCT ACReSS and will not be revealed to any researchers or staff until the end of the enrollment period. As this is a single-blinded trial, all assessments after enrollment will be conducted by independent raters with no knowledge of any treatment and care provided this trial.

### Statistical method

All findings will be reported according to the revised CONSORT statement[36]. All analyses will be performed using SPSS 17.0 J (SPSS Inc., Chicago, IL, USA). All data will be analyzed under the intent-to-treat principle. The primary outcome will be analyzed using a simple Student's *t *test and analyzed regarding potential confounders (e.g., age, gender, sociodemographic factors, site, and clinical characteristics) using regression models. Secondary outcomes will be analyzed using relevant tests at each end point and for possible confounders as described for the primary outcome. Subgroup analysis will be conducted at sites and performed for any possible confounders to differentiate the effectiveness of each situation and explore cluster effects.

## Discussion

This trial has some possible limitations regarding sample size and the duration of case management. Previous studies of EIS for patients with FEP have suggested that although 1.5-year EIS improved patient social functions and readmission rates, these effects did not persist (5 years)[23,24]. However, these results also depend on the treatment provided to the control group, and community mental health services are widely available in Holland and England[1]. In the present situation in Japan, little psychological education is provided in schools and the workplace, and few mental health services are available in the community setting; consequently, much prejudice, discrimination, and stigma exists regarding psychosis[10]. In addition, we will emphasize "graduating from the service" in that case managers will continue to provide services after EIS after a participant enrolls in the trial. Therefore, this trial will demonstrate the effectiveness of EIS in Japan and also explore and consider exit strategies for EIS. Finally, the results of this trial will be used to inform policy makers and practitioners about the benefits, required human resources, and cost-effectiveness of EIS. This trial will provide helpful results about the effectiveness and cost-effectiveness of EIS in Japan to improve the quality and quantity of community mental services.

## Conflict of interest

The authors declare that they have no competing interests.

## Authors' contributions

SK and AN equally contributed to design and management in this trial, and wrote most of the manuscript. SY made substantial contributions to conception and design of this trial and wrote the manuscript with regard to psychosocial care. KI, SM, TN, and HH made substantial contributions to conception and design of this trial. KK, IF, MH, and YO are the directors at each site and made substantial contributions to the revision of the design and management in this trial. All authors read and approved the final manuscript.
